# Hypomagnesemia and clinical benefits of anti-EGFR monoclonal antibodies in wild-type KRAS metastatic colorectal cancer: a systematic review and meta-analysis

**DOI:** 10.1038/s41598-018-19835-8

**Published:** 2018-02-01

**Authors:** Meng-Chiao Hsieh, Chun-Feng Wu, Chun-Wei Chen, Chung-Sheng Shi, Wen-Shih Huang, Feng-Che Kuan

**Affiliations:** 1 Division of Colon and Rectal Surgery, Department of Surgery, Chang Gung Memorial Hospital Chiayi Branch, Chiayi, Taiwan; 2 Graduate Institute of Clinical Medical Sciences, College of Medicine, Chang Gung University, Taoyuan, Taiwan; 3 Centre for Evidence-Based Medicine, Chang Gung Memorial Hospital Chiayi Branch, Chiayi, Taiwan; 4 Department of Hematology and Oncology, Chang Gung Memorial Hospital Chiayi Branch, Chiayi, Taiwan; 5 Department of Gastroenterology and Hepatology, Chang-Gung Memorial Hospital, Taoyuan, Taiwan; 6 Graduate Institute of Clinical Medical Sciences, College of Medicine, Chang-Gung University, Taoyuan, Taiwan; 7 College of Medicine, Chang Gung University, Taoyuan, Taiwan

## Abstract

Hypomagnesemia is a recognized side-effect of cetuximab- or panitumumab-based chemotherapy for metastatic colorectal cancer (mCRC). The clinical relevance of hypomagnesemia is under debate. Thus, a systematic review and meta-analysis of retrospective studies and randomized clinical trials (RCTs) comparing hypomagnesemia with normal magnesium levels in wild-type KRAS mCRC was performed. One RCT, two retrospective studies, and two American Society of Clinical Oncology (ASCO) and European Society for Medical Oncology (ESMO) conference presentations from phase III RCTs involving 1723 patients were included in this study. Patients with hypomagnesemia demonstrated better progression-free survival (PFS) (Hazard ratio [HR]: 0.64; 95% confidence interval [CI]: 0.47–0.88), overall survival (OS) (HR: 0.72; 95% CI: 0.53–0.92), and objective response rate (ORR) (Risk ratio [RR]: 1.81; 95% confidence interval [CI]: 1.30–2.52). By subgroup analysis, frontline, later lines or combination therapy with hypomagnesemia were associated with PFS benefits (HR: 0.78; 95% CI: 0.62–0.98; HR: 0.60; 95% CI: 0.40–0.90; HR: 0.62; 95% CI: 0.41–0.94, respectively). In patients with wild-type KRAS mCRC, hypomagnesemia is associated with better clinical benefits of PFS, OS and ORR when treated with cetuximab- or panitumumab-based chemotherapy. Future clinical trials should corroborate its predictive role.

## Introduction

Personalized medicine has an important role in the treatment of colorectal cancer, which accounts for 8.5% of all cancer-related deaths worldwide^1^. The activation of the epidermal growth factor receptor (EGFR) and downstream signaling of the Ras-Raf-MAP, PI3K, and Akt pathways are associated with the promotion of tumor growth, invasion, metastases, and the inhibition of apoptosis^2–4^. Cetuximab and panitumumab are monoclonal antibodies (MoA) that target the extracellular domain of EGFR and provide survival benefits in metastatic colorectal cancer (mCRC)^5–8^. The mutated Ras gene, mostly seen in the Kirsten rat sarcoma viral oncogene (KRAS) with point mutations in codon 12 and 13 of exon 2, leads to constitutive activation and confer resistance against these biologic agents^9,10^. Although KRAS mutation is a negative predictive marker, the wild-type KRAS does not always guarantee clinical benefits with anti-EGFR MoAs^4,11–13^. Besides, there are proposed positive predictive markers in patients with wild-type KRAS, such as EGFR amplification and overexpression of EGFR ligands^14–16^. Through both negative and positive selection that makes it possible to define the population that benefits more from these anti-EGFR MoAs.

Hypomagnesemia is an appreciated side effect of cetuximab and panitumumab treatment, as reported in randomized trials and meta-analysis^17–20^. Though magnesium is essential to organism, how hypomagnesemia relates to efficacy of these anti-EGFR MoAs and what effect magnesium levels have on cancer progression is an uncertain area of research with contradicting results^21–26^. Whether hypomagnesemia is associated with superior or inferior outcomes remains equivocal in these retrospective or prospective clinical trials. To elucidate the role and clinical benefits of hypomagnesemia in wild-type KRAS mCRC, a systematic review and meta-analysis of current clinical trials was performed.

## Methods

### Data source and search strategy

This study involved a comprehensive search of all published eligible studies and conference presentations that reported the PFS, OS, and ORR for wide-type KRAS mCRC patients with hypomagnesemia after treatment with cetuximab- or panitumumab-based chemotherapy. The searched included MEDLINE, EMBASE, and the Cochrane Central Register of Controlled Trials (CENTRAL) for relevant trials from inception to 01 April 2017. The search strategy were “(hypomagnesemia OR magnesium reduction) AND (cetuximab OR panitumumab OR EGFR) AND (survival OR outcome OR efficacy) AND (colon cancer OR rectal cancer OR colorectal cancer)” without restrictions for language and gender. To identify unpublished studies, the US National Institutes of Health trials register (http://clinicaltrial.gov) and conference abstracts from proceeding of the American Society of Clinical Oncology (ASCO) and the European Society for Medical Oncology (ESMO) were also searched. The ethical approval was waived because all analyses were based on previous published studies.

### Inclusion and exclusion criteria

Studies or conference presentations that investigated wild-type KRAS mCRC treated with cetuximab- or panitumumab-based chemotherapy were included. Those with available data for the events and incidences of hypomagnesemia and those with available Hazard ratio (HR), 95% confidence interval (CI), or Kaplan-Meier survival analysis that compared outcomes in the hypomagnesemia and normal magnesium level groups were also included. The references from included trials were also checked to identify relevant trials. Studies that did not meet these criteria were excluded.

### Data extraction and quality assessment

Two authors independently extracted the available data from the included trials using a standardized data collection form: first author, year of publication or conference presentation, study design, intervention type, study population per group, clinical setting (type of chemotherapy, definition of hypomagnesemia), and outcome data (PFS, OS, and ORR).

Hypomagnesemia was defined according to the CTCAE (Common Terminology Criteria for Adverse Events) or magnesium reduction level^27^. Grade 1 hypomagnesemia was from the lower limit of normal to 1.2 mg/dL, Grade 2 was <1.2 to 0.9 mg/dL, Grade 3 was <0.9 to 0.7 mg/dL, and Grade 4 was <0.7 mg/dL. Those with grades 1–4 hypomagnesemia or > 50% magnesium reduction was arbitrarily classified as the hypomagnesemia group. Observed differences in ORR that could also predict clinically relevant therapeutic benefits and evaluations were done in most trails, according to the RECIST (Response Evaluation Criteria in Solid Tumours) guidelines^28^.

A third author resolved discrepancies in opinion. The quality of methodology was assessed using the Cochrane Collaboration tool for randomized controlled trials (RCTs) and methodological index for non-randomized studies (MINORS)^29,30^.

## Statistical analysis

The Review Manager 5.3 (The Nordic Cochrane Centre, The Cochrane Collaboration, 2014) was used for meta-analysis. The HRs and 95% CIs were extracted for all included trials and subgroups. Data from independent assessment of PFS and OS were pooled together for time-to-event outcomes analysis when both types of reviews were available. A random-effects (RE) model was used to calculate pooled HRs, 95% CIs, and *p* values^31^. A generic inverse variance (IV) meta-analysis was analyzed using log HRs and standard errors (SEs), estimated as described in the Cochrane handbook for systemic reviews of intervention, if the HRs is quoted in a trial together with a 95% CIs or *p* values^30^. If the HRs, 95% CIs, and *p* values were not reported, the HRs and SEs were estimated from Kaplan-Meier survival curve, patient number, censored data, or number at risk table reported^32^. For ORR analysis, risk ratio (RR) and 95% CI for binomial data and dichotomous meta-analysis were calculated with Mantel-Haenszel test under the random-effect model. A two-sided *p* < 0.05 was set as statistically significant.

For heterogeneity tests, the chi-square (chi^2^) and *I*^2^ inconsistency statistics were used^33,34^. A *p* < 0.10 was considered significant heterogeneity. Moreover, *I*^2^ values of 0–24.9%, 25–49.9%, 50–74%, and 75–100% were considered as none, low, moderate, and high heterogeneity, respectively^33,34^.

### Data availability

All data generated or analyzed during this study are included in these published article or unpublished meeting abstracts (ref ^22–26^ with DOI or web link below).

22. 10.1016/S0959-8049(11)71740–3

23. 10.1093/annonc/mdq550

24. 10.1093/annonc/mds577

25. http://meetinglibrary.asco.org/content/140232-158

26. 10.1007/s00280-016-3039-1

## Results

### Eligible studies

**Figure 1:**
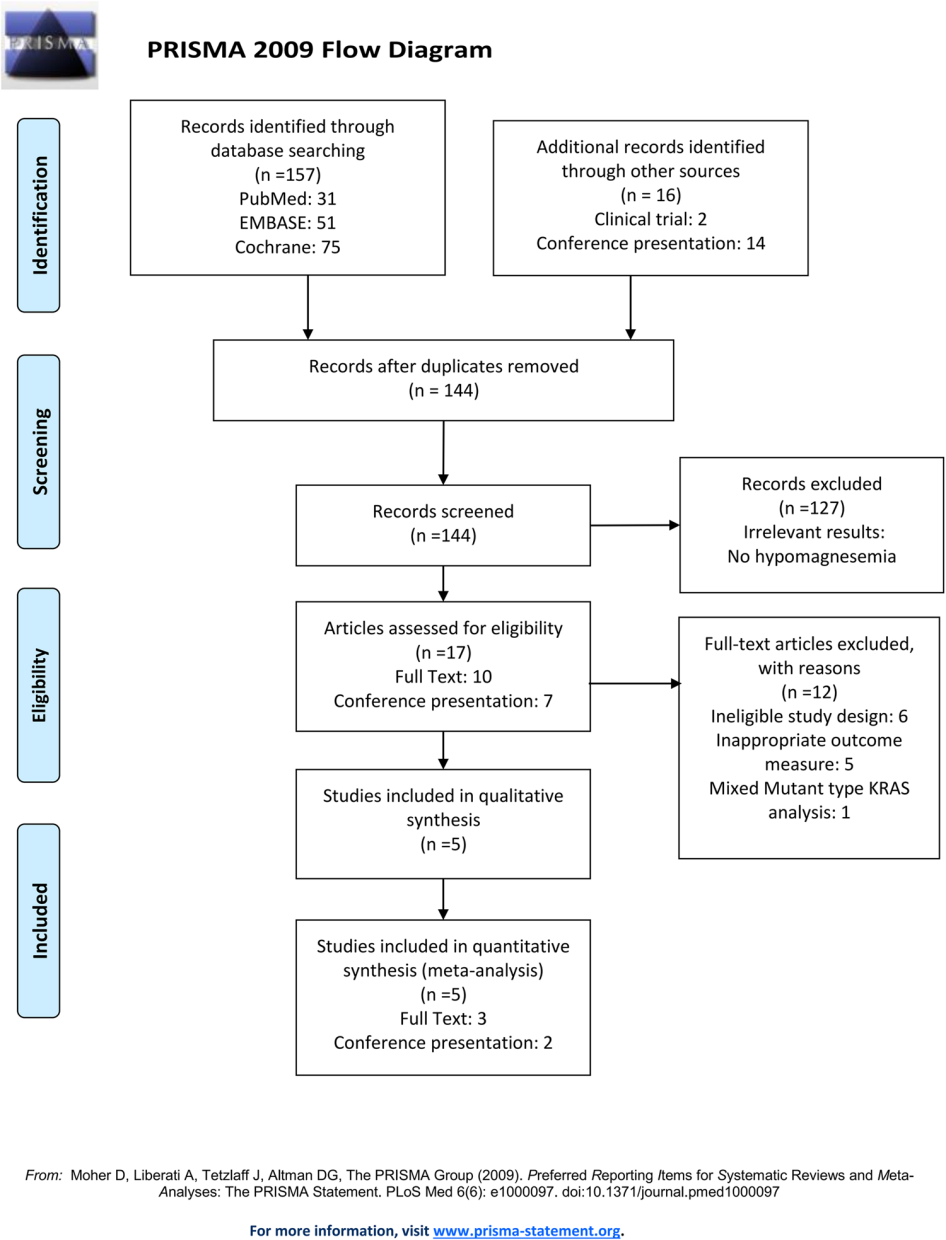
The PRISMA (Preferred Reporting Items for Systematic Reviews and Meta-Analyses) flow diagra^35^.

The PRISMA study flow diagram were shown in Fig. 1^35^. A total of 157 records were identified after searching the MEDLINE, EMBASE, and CENTRAL databases. Another 16 records were identified from the United States National Institutes of Health trials register databases and from conference abstracts of ASCO and ESMO. After removing 29 duplicates and 139 exclusions due to irrelevant results of hypomagnesemia, lack of placebo, or mixed mutant type KRAS analysis, only five trials were incorporated in this meta-analysis. These were two conference presentations of phase III RCTs^22,25^, two retrospective studies^23,26^ and one phase III RCT^24^.

The original setting of CO. 17^5,6^, PRIME^7^ and ASPECCT^8^ were multiple-center and open label phase III RCTs that had a low risk of bias when appraised using the Cochrane Collaboration’s tool^36^. As two non-randomized control trial, Vincenzi *et al*.^23^ and Fujii *et al*.^26^, also had a low risk of bias when appraised using the MINORS appraisal tool^29^.

The subgroup PFS and OS analyses of cetuximab-based chemotherapy were reported by Vincenzi *et al*., CO.17, and ASPECCT, while panitumumab-based chemotherapy was reported by PRIME and ASPECCT. The patients in the Fujii’s trial contained either cetuximab-based or panitumumab-based chemotherapy which analyzed PFS outcome. All trials, except CO. 17, reported ORR. The characteristics of the included trials were summarized in Table 1.

**Table 1:** Characteristics of included trials for meta-analysis.

Author	Trial name	Type of study	Study design	Definition of hypomagnesemia	No. of Patients (Hypo-mg/ Normo-mg)
Fujii *et al*.^26^	N/A	Retrospective	Cetuximab or Panitumumab with mFOLFOX or FOLFIRI vs. mFOLFOX or FOLFIRI in the first-line treatment of mCRC	Grade 0 vs. grade 1–4 hypomagnesemia	14/29
Vincenzi *et al*.^23^	N/A	Retrospective	Cetuximab (400 mg/m2 loading dose, then 250 mg/m2 weekly) with irinotecan in the third-line treatment of mCRC	> 50% vs. < 50% reduction compared with basal value within 1 months from treatment	95/48
Vickers *et al*.^24^	NCIC CTG/ AGITG CO. 17	RCT	Cetuximab (400 mg/m2 loading dose, then 250 mg/m2 weekly) with BSC vs. BSC alone in the third-line treatment of mCRC	Grade 0 vs. grade 1–4 hypomagnesemia	53/163
Price *et al*.^25^	ASPECCT	Conference presentation from phase III RCT	Panitumumab (6 mg/kg every 2 weeks) vs. Cetuximab (400 mg/m2 loading dose, then 250 mg/m2 weekly) in the third-line treatment of mCRC*	Grade 0 vs. grade 1–4 hypomagnesemia	Cetuximab group: 95/408 Panitumumab group:
					143/353
Burkes *et al*.^22^	PRIME	Conference presentation from phase III RCT	Panitumumab (6 mg/kg every 2 weeks) with FOLFOX4 vs. FOLFOX4 alone in the first-line treatment of mCRC	Grade 0 vs. grade 1–4 hypomagnesemia	168/154

**Hypo-mg:** hypo-magnesemia, **Normo-mg:** normo-magnesemia, **FOLFOX:** folinic acid, fluorouracil& oxaliplatin, **FOLFIRI:** folinic acid, fluorouracil& irinotecan, **mCRC:** metastatic colorectal cancer, **NCIC CTG/AGITG CO. 17:** National Cancer Institute of Canada Clinical Trials Group and Australasian Gastrointestinal Trials Group CO. 17, **RCT:** randomized control trial, **BSC:** best supportive care, **ASPECCT:** A Study of Panitumumab Efficacy and Safety Compared to Cetuximab, **PRIME:** Panitumumab Randomized Trial in Combination with Chemotherapy for Metastatic Colorectal Cancer to Determine Efficacy.

*The panitumumab and cetuximab arm in ASPECCT was separated for analysis.

### Analysis of hypomagnesemia and PFS benefit

**Figure 2:**
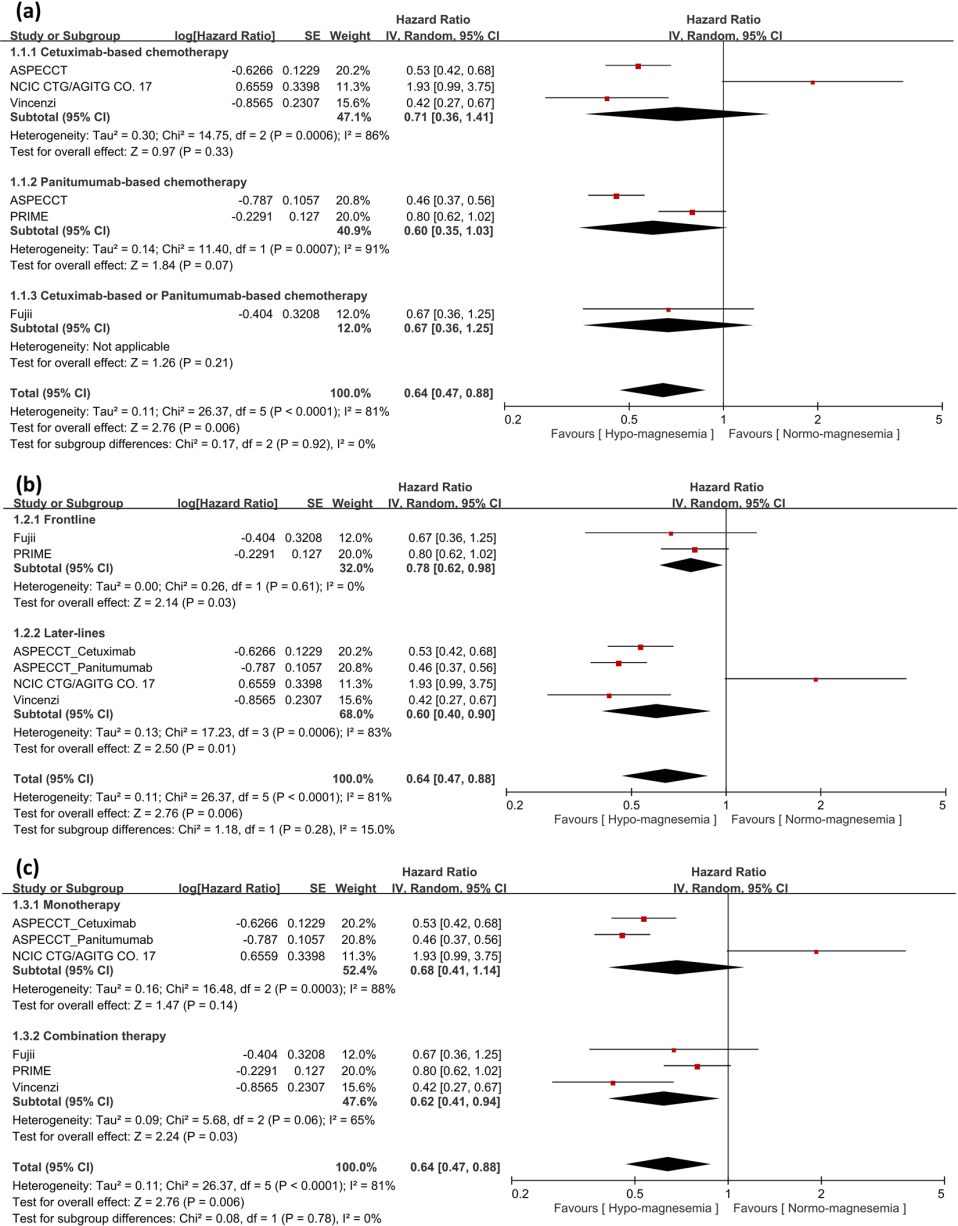
Analysis of progression-free survival (PFS) benefit in patients with hypomagnesemia or normo-magnesemia. (**a**) Analysis of PFS in terms of cetuximab- or panitumumab-based chemotherapy. Pooled analysis showed significant PFS benefit in patients with hypomagnesemia. (**b**) Analysis of PFS in terms of frontline or later-lines of anti-EGFR MoA-based chemotherapy revealed significant PFS benefit in both frontline and later-lines therapy. (**c**) Analysis of PFS in terms of anti-EGFR MoA monotherapy or combination therapy revealed significant PFS benefit in combination therapy and a trend for PFS benefit in monotherapy. **Squares**, study-specific hazard ratios (size of the square reflected the study-specific statistical weight); **Horizontal lines**, 95% CIs; **Diamond**, summary hazard ratios estimate with its 95% CI.

There were five trials of 1723 wild-type KRAS mCRC patients who received either cetuximab- or panitumumab-based chemotherapy, including 568 patients (33.0%) with clinical hypomagnesemia and 1155 patients (67.0%) with normal serum magnesium levels. In the hypomagnesemia group, anti-EGFR MoA-based chemotherapy was statistically significantly associated with 36% reduction in the risk of diseases progression or death (HR: 0.64; 95% CI: 0.47-0.88; *p* = 0.006) (Fig. 2a). After frontline or later-lines of anti-EGFR MoA-based chemotherapy, patient with hypomagnesemia gained significant PFS benefit (frontline, HR: 0.78; 95% CI: 0.62-0.98; *p* = 0.03 and later-lines, HR: 0.60; 95% CI: 0.40–0.90; *p* = 0.01) (Fig. 2b). Subgroup analysis of hypomagnesemia after anti-EGFR MoA monotherapy or combination therapy also showed significant PFS benefit in combination therapy (HR: 0.62; 95% CI: 0.41–0.94; *p* = 0.03) and a trend for PFS benefit in monotherapy (HR: 0.68; 95% CI: 0.41–1.14; *p* = 0.14) (Fig. 2c).

### Analysis of hypomagnesemia and OS benefit

**Figure 3:**
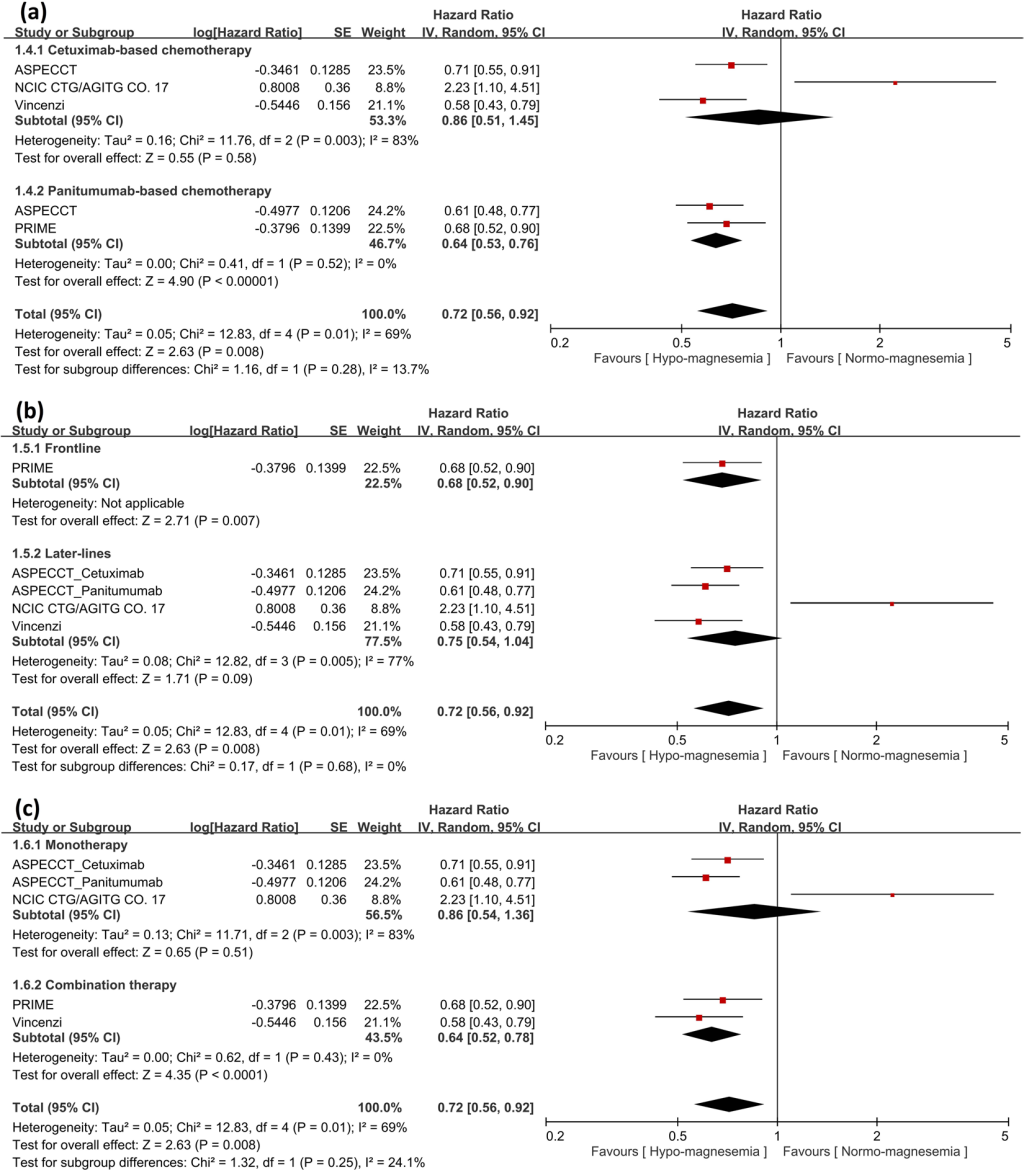
Analysis of overall survival (OS) benefit in patients with hypomagnesemia or normo-magnesemia. (**a**) Analysis of OS in terms of cetuximab- or panitumumab-based chemotherapy. Pooled analysis showed significant OS benefit in patients with hypomagnesemia. (**b**) Analysis of OS in terms of frontline or later-lines of anti-EGFR MoA-based chemotherapy revealed significant OS benefit in frontline therapy and a trend for OS benefit in later-lines therapy. (**c**) Analysis of OS in terms of anti-EGFR MoA monotherapy or combination therapy revealed significant OS benefit in combination therapy and a trend for OS benefit in monotherapy.

Fujii *et al*. did not report OS and only four trials were included in this meta-analysis. There was also significant OS benefit in patients with hypomagnesemia after anti-EGFR MoA-based chemotherapy (HR: 0.72; 95% CI: 0.56–0.92; *p* = 0.008) (Fig. 3a). Subgroup analysis of patients with hypomagnesemia after panitumumab-based chemotherapy revealed significant OS benefit (HR: 0.64; 95% CI: 0.53–0.76; *p* < 0.001). However, patients with hypomagnesemia after cetuximab-based chemotherapy had no OS benefit (HR: 0.86; 95% CI: 0.51–1.45; *p* = 0.58). Subgroup analysis of patients with hypomagnesemia after frontline anti-EGFR MoA-based chemotherapy revealed significant OS benefit (frontline, HR: 0.68; 95% CI: 0.52–0.90; *p* = 0.007 and later-lines, HR: 0.75; 95% CI: 0.54–1.04; *p* = 0.09) (Fig. 3b). Subgroup analysis of patients with hypomagnesemia after combination therapy showed significant OS benefit (combination therapy, HR: 0.64; 95% CI: 0.52–0.78; *p* < 0.001 and monotherapy, HR: 0.86; 95% CI: 0.54–1.36; *p* = 0.51) (Fig. 3c).

### Analysis of hypomagnesemia and ORR benefit

**Figure 4:**
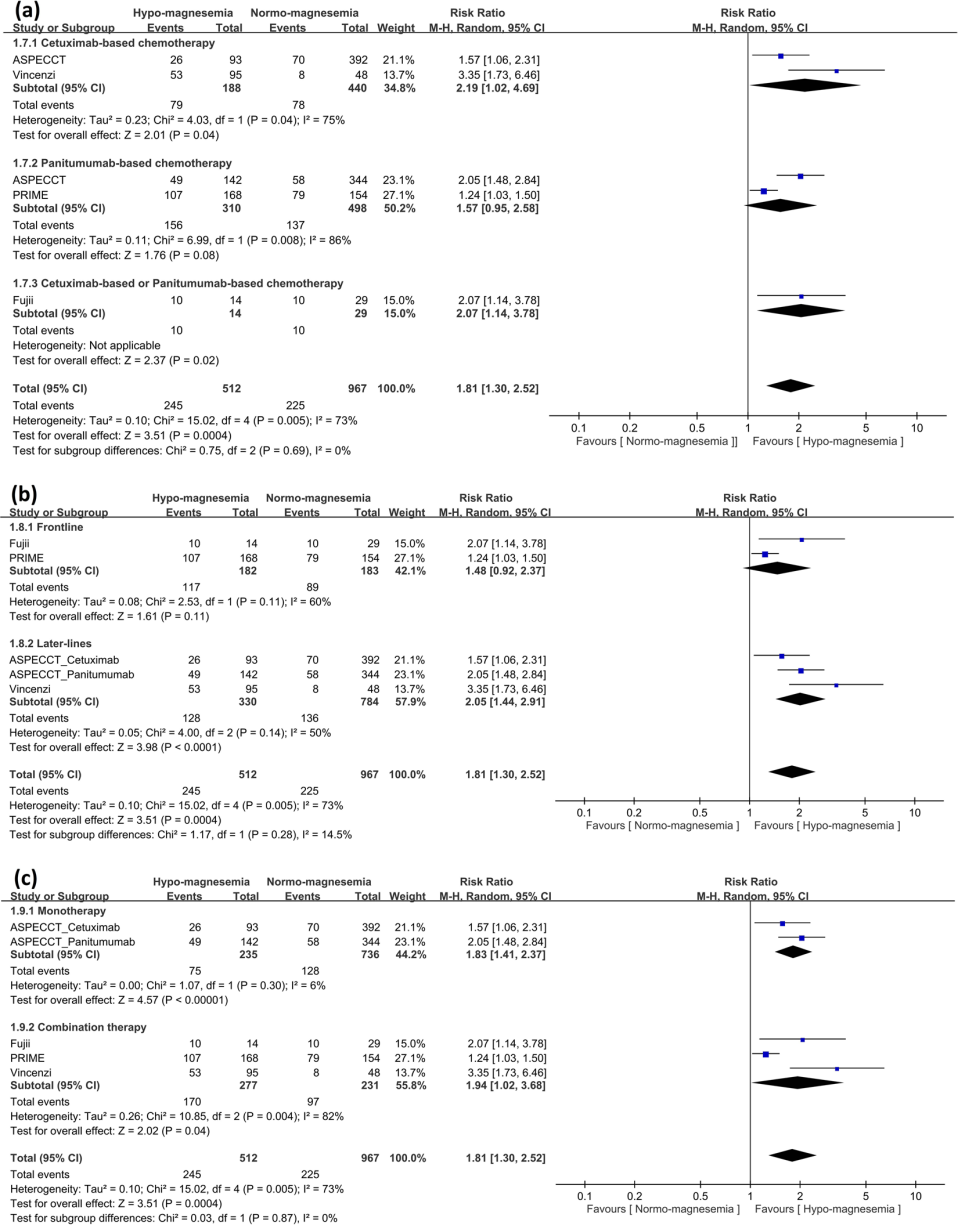
Analysis of objective response rate (ORR) benefit in patients with hypomagnesemia or normo-magnesemia. (**a**) Analysis of ORR in terms of cetuximab- or panitumumab-based chemotherapy. Pooled analysis showed significant ORR benefit in patients with hypomagnesemia. (**b**) Analysis of ORR in terms of frontline or later-lines of anti-EGFR MoA-based chemotherapy revealed significant ORR benefit in later-lines therapy and a trend for OS benefit in frontline therapy. (**c**) Analysis of ORR in terms of anti-EGFR MoA monotherapy or combination therapy revealed significant ORR benefit in both monotherapy and combination therapy.

**Figure 5:**
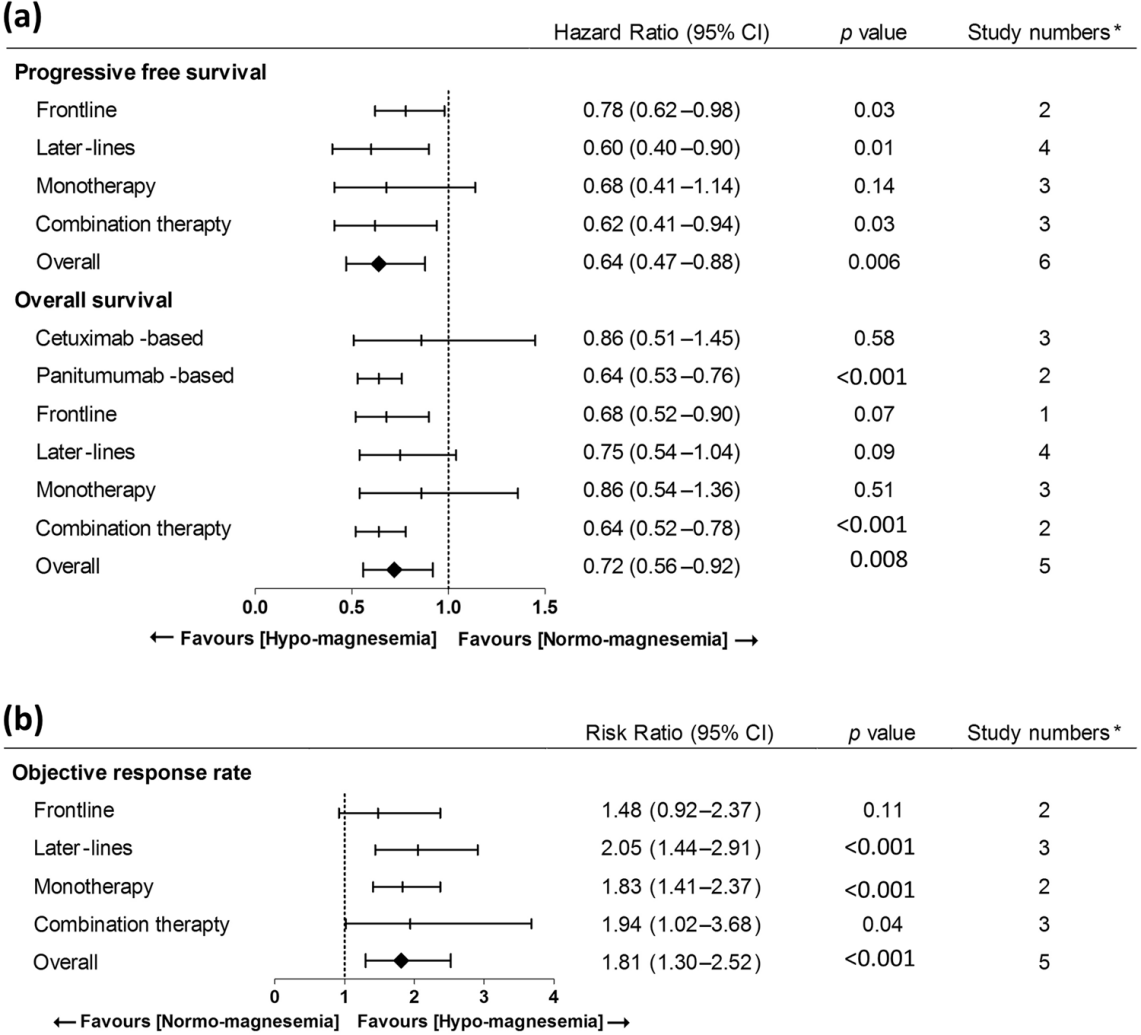
Summary of Findings (*The panitumumab and cetuximab arm in ASPECCT was separated for analysis) (**a**) Analysis of PFS and OS in terms of cetuximab- or panitumumab-based chemotherapy. (**b**) Analysis of ORR in terms of cetuximab- or panitumumab-based chemotherapy.

The CO. 17 trial did not report ORR and only four trials were included in this meta-analysis. In patients with hypomagnesemia, anti-EGFR MoA-based chemotherapy had significantly better ORR (RR: 1.81; 95% CI: 1.30 to 2.52; *p* < 0.001) (Fig. 4a). Moreover, patients with hypomagnesemia irrespective of later-lines of anti-EGFR MoA-based chemotherapy had significant ORR (frontline, RR: 1.48; 95% CI: 0.92–2.37; *p* = 0.11 and later-lines, HR: 2.05; 95% CI: 1.44–2.91; *p* < 0.001) (Fig. 4b). Subgroup analysis revealed significant ORR benefit in anti-EGFR MoA monotherapy and combination therapy (monotherapy, HR: 1.83; 95% CI: 1.41–2.37; *p* < 0.001 and combination therapy, HR: 1.94; 95% CI: 1.02–3.68; *p* = 0.04) (Fig. 4c). The HRs or RRs for all of the different comparisons were summarized in Fig. 5.

## Discussion

The KRAS mutation is a deeply-rooted negative predictive marker in mCRC treated with cetuximab or panitumumab^9,10^. Recently, extended RAS mutation (in addition to exons 3 and 4 of KRAS and exons 2, 3, 4 of neuroblastoma RAS viral oncogene [NRAS]) is proposed as an even more potent negative predictive marker^13^. Stepwise strategy with negative^4,11–13^ and positive selection^14–16^ seems to help define the most benefit for anti-EGFR MoAs, but not simply “opt-in” strategy in patients with wild-type KRAS tumors. This study suggests that hypomagnesemia in patients with wild-type KRAS is associated with better PFS (HR: 0.64; 95% CI: 0.47–0.88), OS (HR: 0.72; 95% CI: 0.56–0.92), and ORR (RR: 1.81; 95% CI: 1.30–2.52) when treated with cetuximab- or panitumumab-based regimen. Previous literatures only indicate the risk and incidence of hypomagnesemia in anti-EGFR MoAs, but not its relationship with clinical efficacy, such as PFS, OS and ORR in this study^17–20^. In addition, serum magnesium levels are easy to check and follow-up during active treatment. Thus, it is useful in decision-making for tailor-made treatment. Skin rash, another well-documented adverse effect of anti-EGFR MoAs, is reportedly predictive of better clinical benefits in mCRC patients^37^. However, anti-EGFR MoAs are not beneficial in patients with mutated KRAS and in clinical practice, the severity of skin rash may be difficult to document among different observers.

Subgroup analysis reveals that panitumumab has a trend for PFS (panitumumab, HR: 0.60; 95% CI: 0.35–1.03 and cetuximab, HR: 0.71; 95% CI: 0.36–1.41) and significant OS (panitumumab, HR: 0.64; 95% CI: 0.53–0.76 and cetuximab, HR: 0.86; 95% CI: 0.51–1.45) benefits in patients with hypomagnesemia. On the other hand, cetuximab had significant ORR (cetuximab, RR: 2.19; 95% CI: 1.02–4.69 and panitumumab, RR: 1.57; 95% CI: 0.95–2.58). The differences of these two anti-EGFR MoAs mainly resulted from the negative impact of CO.17 on PFS and OS^24^. Although they performed the analyses in a phase III trial, RCT CO.17, substantial cases (10.6%) were excluded due to missing serum magnesium levels and KRAS status compared to the ASPECCT or PRIME trials (<2.8%)^22,25^. Thus, it is immature here to interpret the differences of these two anti-EGFR MoAs in view of hypomagnesemia and clinical efficacy.

Nonetheless, cetuximab and panitumumab can lead to hypomagnesemia in varying extent^18,20^. Although the mechanism of hypomagnesemia may lie in the interaction of anti-EGFR MoA, with transient receptor potential cation channel, subfamily M, and member 6 (TRPM6) in the intestine and distal collecting tubules, there is little known about the difference between cetuximab and panitumumab, such as immunogenic properties, half-life, and dosing intervals^38^. Analyses of frontline, later-lines, monotherapy, and combination therapy suggest that cetuximab and panitumumab may possess different properties related to hypomagnesemia and efficacy. The underlying mechanism of hypomagnesium and this favorable response toward cancer treatment is lacking and not well elucidated^21^, and it is inferred that hypomagnesemia induces upregulation of EGFR ligands as magnesiotropic hormone, which in turn, makes tumor more susceptible to anti-EGFR MoAs^14,16,39^. With diverging roles of magnesium in tumor biology, careful preclinical models are awaited to demonstrate this underlying mechanism.

This study has some limitations. First, not all included trials are RCTs and some are non-full-published meeting posters or abstracts^22,25^. However, CO.17, PRIME, and ASPECCT are randomized, controlled, phase III trials in original design^5–8^. Future full publications with unpublished data are likely to be relatively modest and are unlikely to substantively change the results reported here. In addition, the trials included here are not based on an individual patient data (IPD) but abstracted data from published literatures^40^. Despite attempts to contact the authors for original data, data from the studies by Vickers *et al*. and Vincenzi *et al*. are missing. Regulatory authorities should play an important role in support such an IPD meta-analysis and re-evaluate the extent of hypomagnesemia and its predictive role in anti-EGFR MoAs. Funnel plots, Egger’s test and sensitivity analysis were not performed for each pairwise comparison due to less than 10 studies included in this meta-analysis. Instead, all available studies were enrolled for analysis. Lastly, hypomagnesemia is associated with better outcomes in patients with the wild-type KRAS mCRC in this study, but this warrants further validation in extended RAS mutation population. Prospectively designed study is needed to elucidate the magnitude of hypomagnesemia and its predictive role on cetuximab and panitumumab, and how magnesium supplementation can influence these outcomes.

## Conclusions

In patients with wild-type KRAS mCRC, hypomagnesemia is associated with better clinical benefits of PFS, OS and ORR when treated with cetuximab- or panitumumab-based chemotherapy. Future clinical trials should corroborate its predictive role.

## References

[1] GLOBOCAN (2012) *Country Fast Stat*. Available at http://globocan.iarc.fr/Pages/fact_sheets_cancer.aspx (assessed on 9 May 2017).

[2] Spano JP (2005). Epidermal growth factor receptor signaling in colorectal cancer: preclinical data and therapeutic perspectives. Ann Oncol..

[3] Heinemann V, Stintzing S, Kirchner T, Boeck S, Jung A (2009). Clinical relevance of EGFR- and KRAS-status in colorectal cancer patients treated with monoclonal antibodies directed against the EGFR. Cancer Treat Rev..

[4] Bertotti A (2015). The genomic landscape of response to EGFR blockade in colorectal carcinoma. Nature..

[5] Jonker DJ (2007). Cetuximab for the treatment of colorectal cancer. N Engl J Med..

[6] Van Cutsem E (2011). Cetuximab plus irinotecan, fluorouracil, and leucovorin as first-line treatment for metastatic colorectal cancer: updated analysis of overall survival according to tumor KRAS and BRAF mutation status. J Clin Oncol..

[7] Douillard JY (2014). Final results from PRIME: randomized phase III study of panitumumab with FOLFOX4 for first-line treatment of metastatic colorectal cancer. Ann Oncol..

[8] Price TJ (2014). Panitumumab versus cetuximab in patients with chemotherapy-refractory wild-type KRAS exon 2 metastatic colorectal cancer (ASPECCT): a randomized, multicentre, open-label, non-inferiority phase 3 study. Lancet Oncol..

[9] Normanno N (2009). Implications for KRAS status and EGFR-targeted therapies in metastatic CRC. Nat Rev Clin Oncol..

[10] Siena S, Sartore-Bianchi A, Di Nicolantonio F, Balfour J, Bardelli A (2009). Biomarkers predicting clinical outcome of epidermal growth factor-targeted therapy in metastatic colorectal cancer. J Natl Cancer Inst..

[11] De Roock W (2010). Effects of KRAS, BRAF, NRAS, and PIK3CA mutations on the efficacy of cetuximab plus chemotherapy in chemotherapy-refractory metastatic colorectal cancer: a retrospective consortium analysis. Lancet Oncol..

[12] Rowland A (2015). Meta-analysis of BRAF mutation as a predictive biomarker of benefit from anti-EGFR monoclonal antibody therapy for RAS wild-type metastatic colorectal cancer. Br J Cancer..

[13] Sorich MJ (2015). Extended RAS mutations and anti-EGFR monoclonal antibody survival benefit in metastatic colorectal cancer: a meta-analysis of randomized, controlled trials. Ann Oncol..

[14] Jacobs B (2009). Amphiregulin and epiregulin mRNA expression in primary tumors predicts outcome in metastatic colorectal cancer treated with cetuximab. J Clin Oncol..

[15] Laurent-Puig P (2009). Analysis of PTEN, BRAF, and EGFR status in determining benefit from cetuximab therapy in wild-type KRAS metastatic colon cancer. J Clin Oncol..

[16] Seligmann JF (2016). Combined epiregulin and amphiregulin expression levels as predictive biomarker for panitumumab therapy benefit or lack of benefit in patients with RAS wild-type advanced colorectal cancer. JAMA Oncol..

[17] Cao Y, Liao C, Tan A, Liu L, Gao F (2010). Meta-analysis of incidence and risk of hypomagensemia with cetuximab for advanced cancer. Chemotherapy..

[18] Petrelli F, Borgonovo K, Cabiddu M, Ghilardi M, Barni S (2012). Risk of anti-EGFR monoclonal antibody- related hypomagnesemia: systematic review and pooled analysis of randomized studies. Expert Opin Drug Saf..

[19] Chen P, Wang L, Li H, Liu B, Zou Z (2013). Incidence and risk of hypomagnesemia in advanced cancer patients treated with cetuximab: A meta-analysis. Oncol Lett..

[20] Wang Q (2015). Electrolyte disorders assessment in solid tumor patients treated with anti-EGFR monoclonal antibodies: a pooled analysis of 25 randomized clinical trials. Tumour Biol..

[21] Wolf FI, Cittadini AR, Maier JA (2009). Magnesium and tumors, ally or foe?. Cancer Treat Rev.

[22] Burkes R (2011). Randomized, open-label, phase 3 study of panitumumab (Pmab) with FOLFOX4 vs FOLFOX4 alone as 1st-line treatment for metastatic colorectal cancer (mCRC) – the role of hypomagnesemia (Hypomag) on efficacy. Eur J Cancer..

[23] Vincenzi B (2011). Early magnesium modifications as a surrogate marker of efficacy of cetuximab-based anticancer treatment in KRAS wild-type advanced colorectal cancer patients. Ann Oncol..

[24] Vickers MM (2013). Association of hypomagnesemia with inferior survival in a phase III, randomized study of cetuximab plus best supportive care versus best supportive care: NCIC CTG/AGITG CO.17. Ann Oncol..

[25] Price TJ (2015). Randomized phase 3 study of panitumumab vs. cetuximab in chemo-refractory wild-type KRAS exon 2 metastatic colorectal cancer: outcomes by hypomagnesemia in ASPECCT. J Clin Oncol.

[26] Fujii H (2016). Hypomagnesemia is a reliable predictor for efficacy of anti-EGFR monoclonal antibody used in combination with first-line chemotherapy for metastatic colorectal cancer. Cancer chemother and pharmacol..

[27] Trotti A (2003). CTCAEv3.0: development of a comprehensive gradeing system for the adverse effects of cancer treatment. Semin Radiat Oncol..

[28] Eisenhauer EA (2009). New response evaluation criteria in solid tumors: revised RECIST guideline (version 1.1). Eur J Cancer..

[29] Slim K (2003). Methodological index for non-randomized studies (minors): development and validation of a new instrument. ANZ J Surg..

[30] Higgins, J. P. & Green, S. eds Cochrane collaboration. Cochrane Handbook for Systematic Reviews of Interventions. John Wiley & Sons: West Sussex, England (2008).

[31] DerSimonian R, Laird N (1986). Meta-analysis in clinical trials. Controlled Clin Trials..

[32] Tierney JF, Stewart LA, Ghersi D, Burdett S, Sydes MR (2007). Practical methods for incorporating summary time-to-event data into meta-analysis. Trials..

[33] Higgins JP, Thompson SG (2002). Quantifying heterogeneity in a meta-analysis. Stat Med..

[34] Higgins JP, Thompson SG, Deeks JJ, Altman DG (2003). Measuring inconsistency in meta-analyses. BMJ..

[35] Moher, D., Liberati, A., Tetzlaff, J. & Altman, D. G., PRISMA Group. Preferred reporting items for systematic reviews and meta-analyses: the PRISMA statement. *Ann Intern Med*. **151**(4), 264–269 (2009). 10.7326/0003-4819-151-4-200908180-00135 19622511

[36] Petrelli F, Borgonovo K, Barni S (2013). The predictive role of skin rash with cetuximab and panitumumab in colorectal patients: a systematic review and meta-analysis of published trials. Target Oncol..

[37] Egger M, Davey SG, Schneider M (1997). & Minder, C. Bias in meta-analysis detected by a simple, graphical test. BMJ..

[38] Tejpar S (2007). Magnesium wasting associated with epidermal-growth-factor receptor-targeting antibodies in colorectal cancer: a prospective study. Lancet Oncol..

[39] Groenestegem WM (2007). Impaired basolateral sorting of pro-EGF causes isolated recessive renal hypomagnesemia. J Clin Invest..

[40] Piedbois P, Buyse M (2014). Meta-analyses based on abstract data: a step in the right direction, but only a first step. J Clin Oncol..

